# Recognizing Obesity in Adult Hospitalized Patients: A Retrospective Cohort Study Assessing Rates of Documentation and Prevalence of Obesity

**DOI:** 10.3390/jcm7080203

**Published:** 2018-08-07

**Authors:** Mohammad A. Hossain, Ami Amin, Anju Paul, Huzaif Qaisar, Monika Akula, Alireza Amirpour, Shreya Gor, Sofi Giglio, Jennifer Cheng, Roy Mathew, Tushar Vachharajani, Mohamed Bakr, Arif Asif

**Affiliations:** 1 Department of Medicine, Jersey Shore University Medical Center, 1945 State Route 33, Neptune, NJ 07753, USA; mohammad.hossain@hackensackmeridian.org (M.A.H.); ami.amin@hackensackmeridian.org (A.A.); anju.paul@hackensackmeridian.org (A.P.); huzaif.qaisar@hackensackmeridian.org (H.Q.); monika.akula@hackensackmeridian.org (M.A.); alireza.amirpour@hackensackmeridian.org (A.A.); shreya.gor@hackensackmeridian.org (S.G.); sofigiglio16@gmail.com (S.G.); jennifers.cheng@hackensackmeridian.org (J.C.); mohamed.bakr@hackensackmeridian.org (M.B.); 2 Hackensack-Meridian School of Medicine at Seton Hall University, Hackensack Meridian Health, NJ 07753, USA; 3 Department of Medicine, WJB Dorn VA Medical Center, Columbia, SC 29208, USA; roy.mathew@va.gov; 4 Division of Nephrology, Salisbury VA Health Care System and University of NC, Chapel Hill, NC 27599, USA; tvachh@gmail.com

**Keywords:** obesity, prevalence, documentation, comorbidities

## Abstract

Background: While obesity is a chronic condition that predisposes patients to other more serious disorders, the prevalence and the documentation of obesity as diagnosis has not been extensively studied in hospitalized patients. We conducted a retrospective chart review to investigate the prevalence and documentation of obesity as a diagnosis among patients admitted to our medical center. Method: IRB approval was obtained for this retrospective study. Body mass index (BMI) as per CDC, admission and discharge diagnosis of obesity and common comorbidities (hypertension, diabetes, hyperlipidemia, coronary artery disease, congestive heart disease, chronic kidney disease and chronic obstructive pulmonary disease) were recorded. The length of stay in the hospital was also calculated. We also investigated whether counselling was provided to the obese patients for weight loss. Results: A total of 540 consecutive patients were reviewed with a mean age was 66 ± 6 years. Out of 540 patients only 182 (34%) had normal weight, 188 (35%) of the patients were overweight and 170 (31%) patients were obese. Of the obese group, 55% were female and 45% were male.100 (59%) had class I obesity, 43 (25%) had class II obesity and 27 (16%) class III obesity. Of the obese patients 40/170 (23.5%) patients had obesity documented on the admission problem list and only 21 (12%) had obesity documented as a discharge diagnosis. Only 3 (2%) patients were given appropriate counseling and referral for obesity management during the hospitalization. Comorbidities and their prevalence included, hypertension (68%), diabetes mellitus (35%), hyperlipidemia (36%), coronary artery disease (18%), chronic kidney disease (17%), congestive heart failure (18%) and COPD (24%). The average length of stay in normal weight, overweight and obese patients was similar for all three groups (4.5 ± 0.5 days). Conclusion: A significant number of hospitalized patients were overweight and obese. An overwhelming percentage never had weight status documented. Hospitalization offers health care providers a window of opportunity to identify obesity, communicate risks, and initiate weight management interventions.

## 1. Introduction

Obesity and obesity related disorders, which are associated with multiple comorbidities, have been progressively increasing in the most recent decade but obesity as a medical diagnosis has not been well characterized in the inpatient setting [1,2,3,4,5,6,7]. World Health Organization (WHO) describes obesity as the most visible but most neglected public health problem [3]. Two out of three Americans are considered to be overweight or obese. With increased prevalence, obesity plays a major role in modifying treatment outcomes associated with comorbid chronic diseases [3,5,6,7,8,9,10]. Obesity is associated with other more serious disorders such as diabetes mellitus type 2, hypertension, hyperlipidemia, osteoarthritis, sleep apnea, and many others. Obesity and obesity related disorders impacts our health system by spending approximately $147 billion in medical expenses per year and this number is expected to increase approximately by $1.24 billion per year [3,4].

A recent study conducted in clinics and public hospitals in the United States (USA) demonstrated that the prevalence of overweight and obese individuals among adult outpatients have increased significantly [5,6]. Several studies have reported that obesity has been associated multiple comorbidities [7]. Point of care interventions have been promoted as a means of providing health care services at the moment of contact with any and all health professionals. Despite a positive relationship between the intervention to manage obesity and physician acknowledgement of the issue, obesity seldom appear on a patient’s problem list in the hospital electronic health record. When obesity is recognized on the problem list, there is a greater likelihood of appropriate intervention to manage obesity [8]. A review of the electronic health records in a large health care organization recently found that as many as 65% of recorded body mass indexes (BMI) ≥ 30 were not accompanied with a diagnosis of obesity in the problem list [10].

Due to health care coverage issues and lack of consistent medical coverage for lower socioeconomic groups, emergency departments, urgent care clinics, and inpatient hospital services have become an increasingly important source of primary health care services. One major problem with medical care in such acute care settings is that non urgent medical issues are not consistently addressed. Despite the dramatic increase in the number of obese patient being hospitalized, the weight status of these patient remains underdiagnosed and under documented in the inpatient settings. The purpose of this study was to determine the prevalence of overweight and obesity among hospitalized patients and to assess the extent of documentation of weight status in their inpatient electronic medical records (EMR). The study also assessed the prevalence of chronic conditions associated with obesity to determine the relationship between the comorbid conditions and the BMI (body mass index).

## 2. Methods

The study was conducted at Jersey Shore University Medical Center (a tertiary care hospital) part of Hackensack-Meridian Health in Neptune, New Jersey. EMR was reviewed for all (consecutive) patients admitted under inpatient medical floor during a 60-day period (from September through October 2017). Data extracted from the EMR included age, gender, race, height, weight, BMI, admission diagnosis and discharge diagnosis. Comorbidities associated with obesity (such as hypertension, diabetes mellitus, hyperlipidemia, coronary artery disease, chronic kidney disease, congestive heart failure and chronic obstructive pulmonary disease) were investigated to determine the prevalence in the obese inpatient population. BMI was calculated by the EMR using admission weight and height. Charts were reviewed to ascertain whether counselling was provided to the obese patients for weight loss. BMI was categorized as per Centers for Disease Control and Prevention (CDC) guidelines. Categories were defined as: normal weight (BMI 18–24.9 kg/m^2^), overweight (BMI 25–29.9 kg/m^2^) and obese (BMI > 30 kg/m^2^). The obese group was further divided into 3 classes. Class I (BMI 30–34 kg/m^2^), Class II (BMI 35–39.9 kg/m^2^) and Class III (BMI > 40 kg/m^2^).

Documentation of overweight or obesity status was extracted from EMR, admission and discharge diagnosis lists, admission history notes, physical examination notes and discharge summaries. Patients under 18 years of age and the patients with terminal illness were excluded from the study.

Institutional review board approval was obtained for this study. All study procedures were carried out in accordance with the Declaration of Helsinki regarding research involving human subjects. The summary statistics of continuous variables were reported as mean ± standard deviation. *p* value was considered significant if >0.05. All data analysis was conducted with Microsoft excel software, with means and percentage calculated among subgroups of patients.

## 3. Results

**Figure 1:**
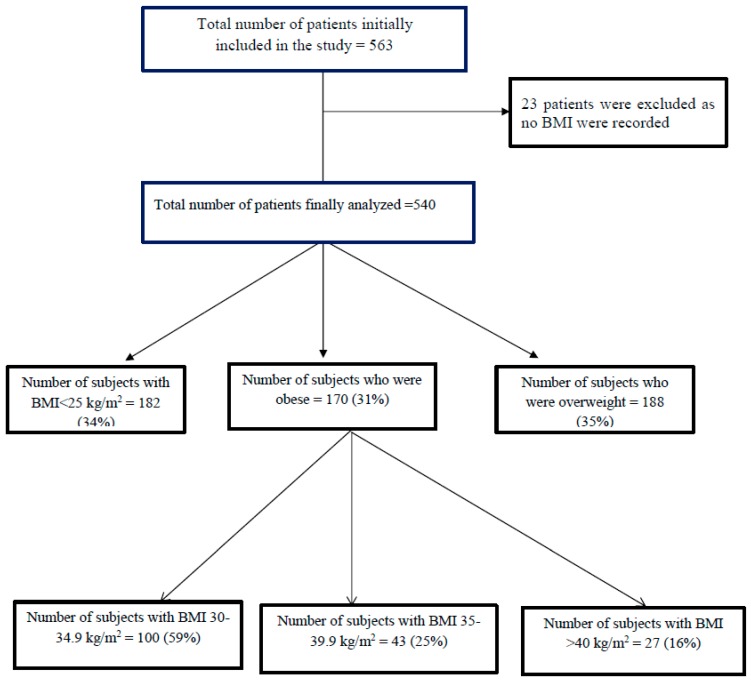
Patient selection.

During the study period, 563 patients were admitted to the medical floor, of those, 540 admission were eligible for the study, and 23 patients were excluded because of the absence of recorded height and BMI. A total of 540 consecutive patients were included in this analysis (Figure 1).

**Figure 2:**
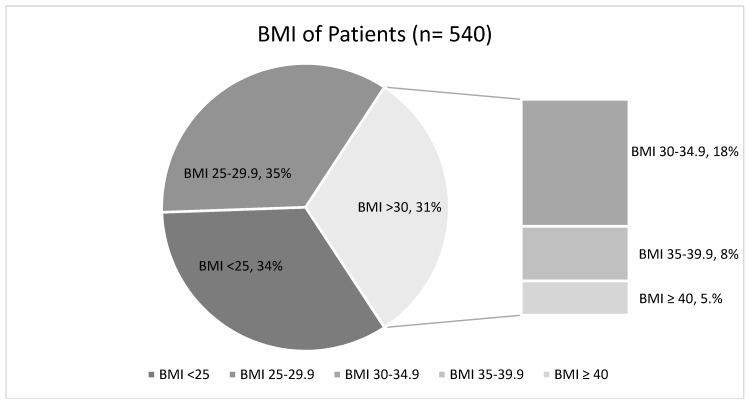
Body mass index (BMI) distribution of the patients (*n* = 540).

Demographic characteristics are shown in Table 1. Patient age ranged from 21–94 years (mean age 66 ± 6 years). Out of 540 patients, only 182 (34%) had normal weight, 188 (35%) of the patients were overweight and 170 (31%) patients were obese (Figure 2). Of the obese group, 100 (59%) had class I obesity, 43 (25%) had class II obesity and 27 (16%) had from class III obesity (Figure 2). Average BMI of all the patients was 28 kg/m^2^. Only 40 (23.5%) patients had obesity documented on the admission problem list and only 21 (12%) had obesity documented as a discharge diagnosis. Only 3 (2%) patients were given appropriate counseling and referral for obesity management during the discharge. Comorbidities and their prevalence included, hypertension (68%), diabetes (35%), hyperlipidemia (36%), coronary artery disease (18%), chronic kidney disease (17%), congestive heart failure (18%) and COPD (24%) (Table 2). There is no significant difference in our cohort. This study also found that diabetes mellitus type 2 (normal weight 15%, overweight 27%, obese 24%; *p* < 0.001) and obstructive sleep apnea (normal weight 2%, overweight 5%, obese 5%; *p* = 0.004) to have significantly increased prevalence in the overweight and obese patients compared to the normal weight. The average length of hospital stay was about 4.5 ± 0.5 days was similar to all three groups (normal weight, overweight and obese patient). The prevalence of obesity was 56% for females and 44% for male patients.

## 4. Discussion

This study found that a significant number (65%) of patients admitted to the hospital for general medical conditions were overweight (34%) and obese (31%). Out of these patients, only 23% had the diagnosis of obesity documented during hospitalization. At the time of discharge, only 21/170 (12%) patients had obesity as a diagnosis on the discharge list. A miniscule percentage (3 of the 170 obese patients) received obesity counseling at the time of discharge highlighting the missed opportunity that could potentially have a positive impact on weight reduction.

The obesity documentation rates of 23% observed in our study are much higher than the obesity documentation rates reported previously in hospitalized patients [11,12,13]. For example, Azhdam et al. noted that only 13.2% of patients in their study had their weight status documented anywhere in their medical records [12]. The same authors found that at the time of discharge less than 1% of hospitalized obese and overweight patients had any documentation of obesity in their discharge summary. Other researchers reported similarly low rates of documentation with only 1.7% of hospitalized obese patients having the diagnosis listed on the discharge summary [13]. Our study found a much higher percentage (12%) of obesity diagnosis at the time of discharge. However, our study still highlights the opportunity gap in appropriately documenting a major chronic disease. An improvement in obesity documentation would allow a focused attention and intervention to reduce obesity.

In our study predominant medical conditions associated with obesity were hypertension (68%), diabetes mellitus (35%), hyperlipidemia (36%), chronic kidney disease (18%), COPD (24%), and coronary artery disease (18%) (Table 2). Multiple previous studies have also documented a high prevalence of chronic diseases in obese patients [3,4,5]. In one study, investigators found a significantly high prevalence of hypertension (18.1% for normal weight and 52.3% for obesity class 3), diabetes mellitus (2.4% for normal weight and 14.2% for obesity class 3) and dyslipidemia (8.9% for normal weight and 19.0% for obesity class 3). We failed to find a significant difference in our cohort (Table 2). We found diabetes (normal weight 15%, overweight 27%, obese 24%; *p* < 0.001) and obstructive sleep apnea (normal weight 2%, overweight 5%, obese 5%; *p* = 0.004) to have significantly increased prevalence in the overweight and obese patients compared to the non-obese patients. There are major differences between our study and the report by Nguyen et al. [4]. The study by Nguyen et al. included general population [4]; however, our study, included only adult hospitalized patients. In addition, Nguyen and colleagues reported on class 3 obesity and the co-morbid conditions. We included both overweight and obese patients. These differences might have led to the discrepancies between the results demonstrated by our study and those reported by Nguyen et al. [4].

The rising financial burden of obesity and obesity related diseases is a global concern and has been a priority for researchers. Reducing direct and indirect cost of obesity can decrease healthcare utilization and demand [3]. Stratifying the cost on obesity into direct and indirect group has focused the healthcare interventions to target the group requiring more concentration [14,15]. One study conducted by Finklestein et al. in 2010 to estimate costs of obesity on a cross sectional basis found that for all categories of obesity, the three variables: healthcare costs, absenteeism, and presentism all increased with increasing BMI [15]. Raising awareness of healthcare providers to help reduce obesity and related complications is a health care priority worldwide [6]. Previous research concluded that documentation of obesity as a medical problem was associated with greater physician attention to patient weight, specifically an increased prevalence of exercise counseling in both inpatient and outpatient settings [8,10,16,17]. In the present study, only 2% patients received obesity related counseling at the time of discharge although 12% patient had obesity diagnosis at discharge summary. Furthermore, adding obesity diagnosis to the problem list and documentation in the medical records increases work RVUs and revenue stream for the facility and providers. Medicare ICD-10 codes allow counseling that has separate reimbursement codes [5,17]. More strategic and policy based study is needed to identify effective methods for documentation, physician counseling and behavioral therapy approaches in the treatment of obesity in both inpatient and primary care settings. We recognize that counselling and drawing attention to problem are not the only measures that lead to a successful outcome [18].

The U.S. preventive service Task Force and the American Academy of Family Physician set guidelines for primary care clinics and recommended that screening for obesity using BMI should be done for all adults [18,19]. There are no clear guidelines to address obesity on the inpatient setting and on how to best interventions to help decrease obesity in the inpatient setting. The most recent recommendation in 2015 from the American College of Cardiology/American Heart Association and the Obesity Society, introduced five major steps for obesity management including identifying those at risk, physician counselling and guidelines for treatment with diet, life style intervention and surgery [20]. The initial step to increasing guideline compliance may be encouraging physicians to document obesity as a medical problem in the patient’s EMR [21]. In our study, only three patients received obesity counseling and lack of recognition is a barrier to care. Clearly, there is a major opportunity that exists in identifying and managing weight status of the patients.

The data from this study also illustrates not only the practices in use but also the types of barriers that may reduce physician effectiveness in weight management. Although provider counseling produce positive result in obesity related behavioral modification, it may be difficult for physician to review BMI in the inpatient settings due to high patient’s census, limited time and high turnover of discharge patients [18]. Given the high prevalence of obesity among hospitalized patients, we believe that the development of hospital-based weight management program may help create an action plan in collaboration with department of endocrinology and metabolism that will mandate BMI calculation for every patient within the electronic medical record and recommend interventions if necessary. The goal for hospital provider and residents should include the documentation of obesity diagnosis to be included in discharge paperwork for further follow up care. The implementation of such a program in a medical center would highlight the importance of weight reduction and make awareness to patients and families. The initial strategies may include nutritional education, cognitive behavioral strategies, emphasis on a supportive family environment, and physical activity prescriptions [22,23]. As a provider, we should accept the responsibility and help to improve the documentation and obesity counselling.

Our study has several limitations. First, retrospective nature and past medical history gleaned from the EMR limits the ability to infer causal effects and limits our analyses to associations. Secondly, the problem list used as a supplement for addressing the problem as part of the clinical encounter because all of the patients admitted for different reason not related to obesity directly. There could have been counseling related to obesity provided by residents and hospitalists that was verbally provided but not documented within the medical records. This may explain very low counseling rate during discharge even in morbidly obese patients. Finally, this is a single-center study and does not reflect the overall picture of other academic hospitals.

## 5. Conclusions

Our study revealed that 31.4% (170 of the 540) patients fit the clinical criteria for obesity but only a small percentage (23.5%) had obesity documented on the admission problem list and even less (12%) had obesity documented on their discharge diagnosis list. Despite recognizing obesity as a problem, only three obese patients were given appropriate counselling during hospitalization. Additionally, obesity was found to be associated with an increase in the prevalence of diabetes mellitus and obstructive sleep apnea. There is a lack of recognition of obesity has a medical problem and even in cases where obesity is recognized, there are missed opportunities for interventions to educate and to engage in interventions for weight reduction. Inpatient admissions offer health care providers a window of opportunity to effectively act on identifying obesity, communicating risks and initiating weight management intervention such as counseling, behavioral and other interventional therapies. We advocate for additional research and quality improvement measures regarding the recognition of obesity as a medical problem and initiation of interventions to help prevent obesity related disorders.

## Figures and Tables

**Table 1:** Demographic characteristic and comorbidities of study population.

	Total	Obese	Overweight	BMI < 25
Number of patients	540	170 (31%)	188 (35%)	182 (34%)
Gender				
Male	237 (44%)	76 (45%)	85 (45%)	76 (42%)
Female	303 (56%)	94 (55%)	103 (55%)	106 (58%)
Race				
Caucasian	420 (78%)	123 (72%)	148 (79%)	149 (82%)
Black	91 (17%)	38 (22%)	27 (14%)	26 (14.2%)
Asian	7 (1%)	2 (1%)	3 (2%)	2 (1%)
Other	19 (3.5%)	6 (3%)	10 (5%)	5 (3%)

**Table 2:** Comorbidities among patients (*n* = 540).

Comorbidities among Patients	Obese (*n* = 170)	Overweight (*n* = 188)	BMI < 25 (*n* = 182)	*p* Value
Hypertension	115 (68%)	116 (62%)	103 (56%)	0.079
Coronary Artery Disease	31 (18%)	52 (27%)	44 (24%)	0.148
Peripheral Vascular Disease	12 (7%)	13 (7%)	8 (4%)	0.47
Chronic Kidney Disease	29 (17%)	34 (18%)	33 (18%)	0.953
Malignancy	24 (14%)	45 (24%)	42 (23%)	0.049
Congestive Heart Failure	88 (16%)	34 (18%)	23 (12.5%)	0.258
Cerebrovascular Accident	66 (12%)	23 (12%)	22 (12%)	0.988
Diabetes Mellitus	139 (24%)	52 (27%)	28 (15%)	<0.001
COPD	113 (21%)	36 (19%)	36 (20%)	0.465
Obstructive Sleep Apnea	28 (5%)	9 (5%)	3 (2%)	0.004
Hypothyroidism	75 (14%)	28 (15%)	23 (12.5%)	0.773
Hyperlipidemia	183 (34%)	67 (35.6%)	54 (29%)	0.321
